# An integrated feature selection approach to high water stress yield prediction

**DOI:** 10.3389/fpls.2023.1289692

**Published:** 2023-12-04

**Authors:** Zongpeng Li, Xinguo Zhou, Qian Cheng, Weiguang Zhai, Bohan Mao, Yafeng Li, Zhen Chen

**Affiliations:** Institute of Farmland Irrigation, Chinese Academy of Agricultural Sciences, Xinxiang, China

**Keywords:** multispectral, PCRF-RFE, Cubist, RNN, performance

## Abstract

The timely and precise prediction of winter wheat yield plays a critical role in understanding food supply dynamics and ensuring global food security. In recent years, the application of unmanned aerial remote sensing has significantly advanced agricultural yield prediction research. This has led to the emergence of numerous vegetation indices that are sensitive to yield variations. However, not all of these vegetation indices are universally suitable for predicting yields across different environments and crop types. Consequently, the process of feature selection for vegetation index sets becomes essential to enhance the performance of yield prediction models. This study aims to develop an integrated feature selection method known as PCRF-RFE, with a focus on vegetation index feature selection. Initially, building upon prior research, we acquired multispectral images during the flowering and grain filling stages and identified 35 yield-sensitive multispectral indices. We then applied the Pearson correlation coefficient (PC) and random forest importance (RF) methods to select relevant features for the vegetation index set. Feature filtering thresholds were set at 0.53 and 1.9 for the respective methods. The union set of features selected by both methods was used for recursive feature elimination (RFE), ultimately yielding the optimal subset of features for constructing Cubist and Recurrent Neural Network (RNN) yield prediction models. The results of this study demonstrate that the Cubist model, constructed using the optimal subset of features obtained through the integrated feature selection method (PCRF-RFE), consistently outperformed the RNN model. It exhibited the highest accuracy during both the flowering and grain filling stages, surpassing models constructed using all features or subsets derived from a single feature selection method. This confirms the efficacy of the PCRF-RFE method and offers valuable insights and references for future research in the realms of feature selection and yield prediction studies.

## 1 Introduction

Winter wheat holds tremendous significance in agriculture as one of the most widely cultivated and consumed food crops across the globe. It plays a pivotal role in providing a substantial portion of food and energy to the world’s population and constitutes an integral part of people’s daily diets (Liu et al., 2023). The production of winter wheat also carries great importance for a nation’s food supply and the overall food security of its citizens (Li et al., 2022). In recent years, the rise in climate-related disasters, marked by frequent extreme weather events such as severe droughts and excessive precipitation, poses a substantial threat to the stability of agricultural yields (King et al., 2015; Lesk et al., 2022; Wu et al., 2022). Therefore, the timely and precise monitoring of crop yields holds the key to empowering farmers and agricultural producers to make well-informed decisions based on yield data. Furthermore, it aids governments and policymakers in comprehending the food supply status and implementing preemptive measures to ensure food security for their populations.

Traditional methods of monitoring crop yields typically necessitate labor-intensive and costly procedures involving destructive sampling and measurements. Moreover, these conventional techniques are constrained by their limited capacity to obtain samples, often leading to incomplete and non-real-time yield data. They can also inadvertently cause soil compaction and damage (Ma et al., 2023). The emergence of drone remote sensing technology has revolutionized agricultural practices. UAV remote sensing offers the capability to swiftly cover vast farmland areas and deliver high-resolution spectral images and data, including multispectral imagery (Su et al., 2023). This innovative technology significantly mitigates long-term operational costs and provides timely, accurate, and comprehensive crop monitoring data, thereby driving progress in the field of agriculture. Multi-spectral sensors, as one of the sensors in UAV remote sensing, are used in yield prediction (Shafiee et al., 2023), environmental monitoring (Lo et al., 2023) and pest and disease monitoring (Barreto et al., 2023). Multispectral sensors are adept at capturing spectral information across multiple bands, typically including the visible and near-infrared ranges, each with distinct reflective characteristics on vegetation. Simultaneously, these sensors can detect finer surface details such as crop rows and vegetation plots.

Drawing from the findings of prior research(Wang et al., 2016; Zheng et al., 2019), it became evident that vegetation indices derived from UAV multispectral imagery exhibited varying performance across different crop types and environmental settings. Hence, it becomes imperative to select these features judiciously to eliminate redundancies. For instance, Zhang et al. (Zhang et al., 2020) employed the PC method within a filtering approach to calculate correlation coefficients between the Absorbance Difference Vegetation Index (ADVI) and conventional vegetation indices and their association with Cu content in soil and leaves. Their findings revealed that ADVI exhibited a significantly stronger correlation compared to other vegetation indices, showcasing its effectiveness in distinguishing between Cu and Pb stress. In a similar vein, Chen et al. (Chen et al., 2022) adopted the random forest importance method as a screening technique within the embedding process to optimize variables for leaf Na^+^ extraction. Their study observed significant enhancements in both models, with the Support Vector Machine (SVM) model emerging as the most effective choice. Leo et al. (Leo et al., 2021) employed RFE within the packing method to choose spectral indices. This led to an improved prediction accuracy for the model when assessing the correlation between predicted and observed scores. It’s noteworthy that all three of the aforementioned feature selection methods have consistently displayed strong performance in previous studies. They have proven to be instrumental in obtaining feature sets that are both reliable and highly effective. Cubist models have found applications in a wide range of fields, including forestry (Ou et al., 2019), soil science (Sarkodie et al., 2023), crop research (Xiao et al., 2020), environmental studies (Azizi et al., 2022), and more. Presently, RNN models have demonstrated strong performance in tasks such as sound activity detection (Mihalache and Burileanu, 2022), image retrieval, natural language processing (Mao et al., 2014), and crop yield prediction (Khaki et al., 2020). Drawing from the collective insights of prior studies, it becomes evident that the majority of research tends to follow one of two approaches. In most cases, studies either exclusively rely on a single feature selection method (Abdel-Rahman et al., 2013; Cabezas et al., 2016), or they engage in comparative analyses involving multiple feature selection methods (Bai et al., 2022; Li et al., 2022). Unfortunately, what’s often missing is the integration and synergistic utilization of diverse feature selection methods to leverage their unique strengths and yield a more rational and dependable subset of features. This, undoubtedly, presents a challenge and an opportunity for further research in our field.

Hence, in this study, we will integrate a combination of feature selection methods, including the filtering method (PC), embedding method (RF importance), and the packing method (RFE), all applied to the selection of features from a pool of 35 multispectral indices. Subsequently, we will employ these different feature subsets to construct Cubist and RNN models for yield prediction. Our aim is twofold: first, to generate feature subsets through the application of various feature selection methods; and second, to establish an integrated feature selection approach that effectively derives these subsets. Ultimately, we intend to evaluate the accuracy of yield prediction models, offering a theoretical foundation for the utilization of multispectral remote sensing in wheat yield monitoring.

## 2 Materials and methods

### 2.1 Experimental area and design

**Figure 1:**
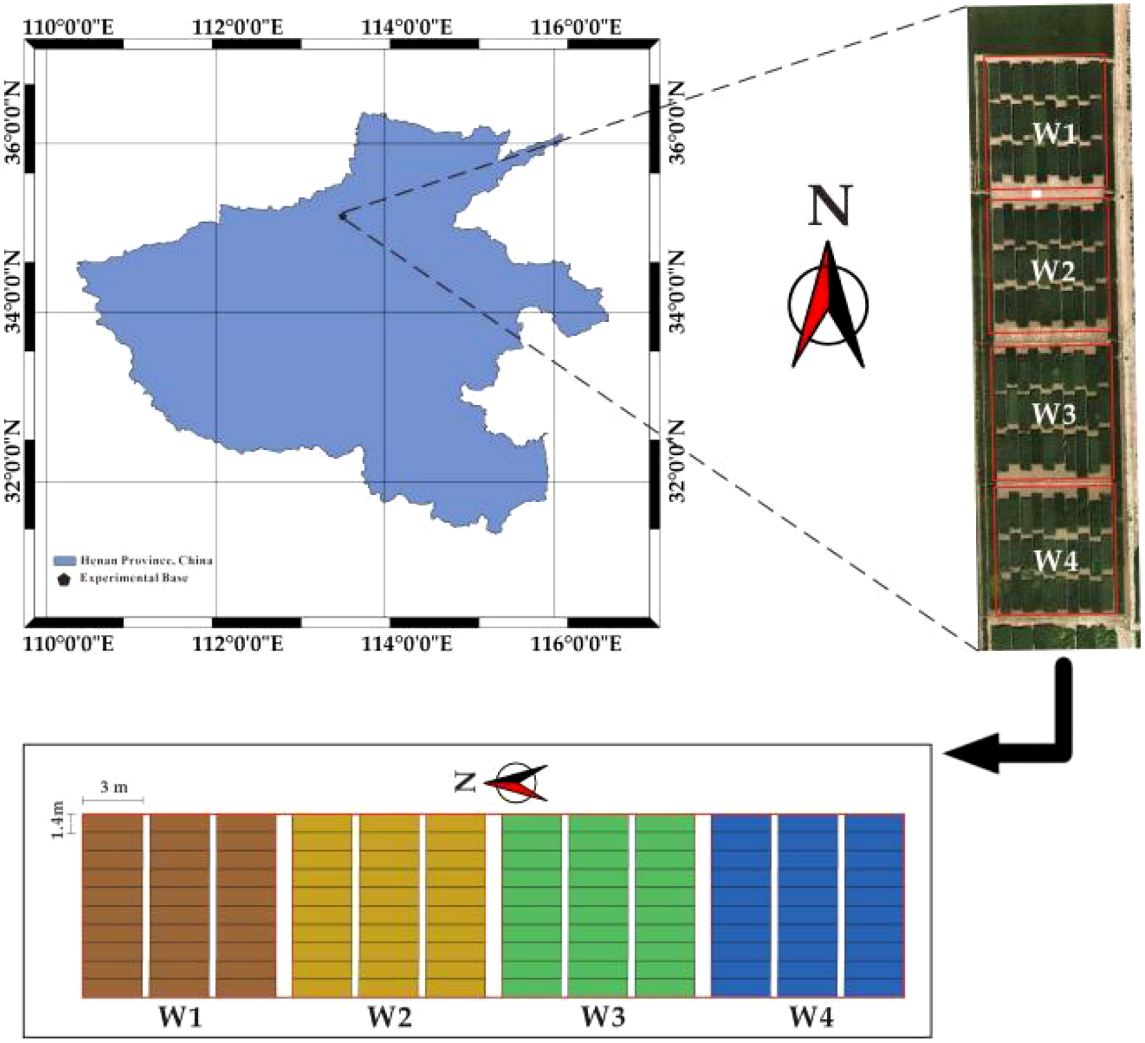
Test area and plots.

This experiment was conducted in 2023 at the Xinxiang Comprehensive Experimental Base of the Chinese Academy of Agricultural Sciences, located in Xinxiang City, Henan Province, at coordinates 113°45′38″E and 35°8′10″N (**Figure 1**), This region boasts extensive plains and a moderate climate that is particularly well-suited for the cultivation of winter wheat. It is recognized as a national high-quality specialty wheat production base.

The experiment consisted of 120 plots. To investigate the impact of extreme weather conditions, specifically extreme rainfall and severe drought, on winter wheat yields in the Xinxiang area, four distinct treatments were established throughout the reproductive stage. These treatments were as follows: low water stress treatment 1 (W1, with 0% full water, 0 mm), low water stress treatment 2 (W2, with 30% full water, 72 mm), full water treatment 3 (W3, with 100% full water, 240 mm), and high water stress treatment 4 (W4, with 200% full water, 480 mm). Each experimental treatment consisted of 30 plots, 3 m long and 1.4 m wide, with an area of 4.2 m^2^ and a row spacing of 20 cm. Ten wheat varieties suitable for cultivation in the Yellow and Huaihe wheat regions were selected as experimental materials. To ensure the objectivity of the experiment, three replications were established for each treatment. The experiment was initiated with planting in early November 2022 and concluded with the harvest on June 10, 2023. In the production area, pesticides and fertilizers are applied in accordance with local management practices. Prior to planting, a basal fertilizer is applied at a rate of 750 kg·ha^-1^. Wheat is sown at a density of 500 plants per acre of land. Subsequently, each plot’s harvested wheat was encapsulated in numbered bags within the laboratory, where it was dried to a constant mass. The weight of winter wheat in each plot was then recorded and converted to yield based on the plot’s area.

### 2.2 UAV multispectral data acquisition

In this experiment, a DJI M210 (Shenzhen DJI Technology Co., Ltd., Shenzhen, China) UAV equipped with a Micasense RedEdgeMX multispectral camera was used to collect multispectral data from winter wheat. The DJI M210 is a high-performance quadcopter known for its exceptional stability and precise flight control. The RedEdgeMX sensor is equipped with five bands: red, green, blue, near-infrared (NIR), and red-edge, making it particularly well-suited for agricultural and environmental monitoring. Each band has a precisely designed center wavelength and bandwidth to meet diverse application requirements. The red band’s center wavelength is 668nm, the green band’s center wavelength is 560nm, the blue band’s center wavelength is 475nm, the NIR band’s center wavelength is 840nm, and the red-edge band’s center wavelength is 717nm. Importantly, each channel offers high resolution at 1280×960 pixels with a wide field of view.

The data collection process involved two flights on May 8, 2023, coinciding with the flowering stage, and May 20, 2023, corresponding to the grain-filling stage. These flights were conducted between 11:00 and 14:00, benefiting from clear and cloudless weather conditions that minimized shadows and ensured optimal image quality. Both UAV flights were maintained at a constant altitude of 30 meters throughout their missions. To enhance data coverage and overlap, a heading overlap rate of 85% and a side overlap rate of 85% were established. These settings were chosen to maximize the comprehensiveness and accuracy of the imagery, ultimately improving the reliability of subsequent data processing and analysis. Moreover, the RedEdgeMX sensor is furnished with Global Navigation Satellite System (GNSS) technology, ensuring millimeter-level accuracy. When coupled with Ground Control Points (GCPs), this technology facilitates precise geo-correction during the post-processing phase. Notably, the sensor operates in the vertical ground equal time interval photo mode, systematically capturing images at regular time intervals. This mode is essential for ensuring uniform spacing of images throughout the flight, enabling the capture of intricate details and changes within the target area.

### 2.3 UAV image preprocessing

**Figure 2:**
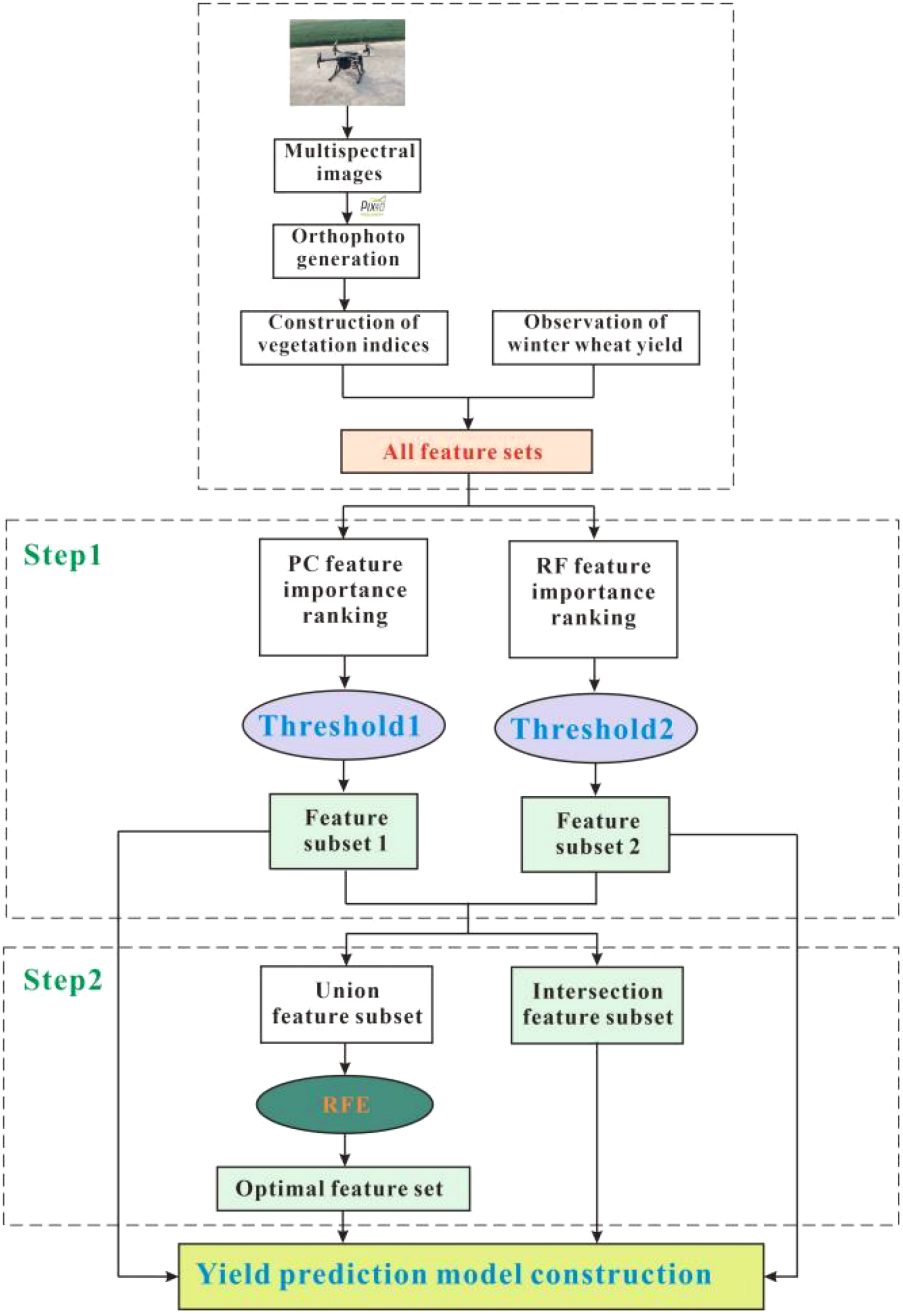
Research framework and flow chart.

The data collected from each of the two stages underwent a systematic process. It commenced with the transfer of the collected data to a computer and its subsequent import into Pix4DMapper Pro software (version 4.4.12) (Pix4D SA, Switzerland), two distinct projects were created, and the necessary project parameters were configured. The alignment of images was achieved through the utilization of the feature point matching algorithm, as illustrated in **Figure 2**. In more detail, the initial step involved generating a sparse point cloud representing the flight area, which was based on the UAV imagery and corresponding position data. Subsequently, a spatial grid was created using the sparse point cloud, and spatial coordinate information was incorporated. This transformation resulted in the creation of a sparse point cloud with precise positions. Following this, the surface geometry of the flight area was generated. Finally, the process culminated in the creation of both a high-resolution digital orthophoto (DOM) and a digital surface model (DSM) encompassing the entire flight area. ArcMap (version 10.8) (Environmental Systems Research Institute, Inc., USA) was used to divide the multispectral high-resolution digital orthophotos into 120 regions with neighbourhood IDs. Using the Zonal Statistics as Table function in the ArcMap software, the average value of each plot was calculated for each of the five bands, and the five spectral bands corresponding to each plot were identified and exported.

### 2.4 Vegetation index acquisition

Drawing from previous research findings, we compiled a comprehensive set of 35 yield-sensitive multispectral vegetation indices. These indices were derived from the acquired reflectance data spanning five bands within the multispectral spectrum. They serve as the primary input feature set for our prediction model. A detailed listing of all the vegetation indices utilized in this study is provided in **Table 1**, including simple vegetation indices, modified vegetation indices, and functional vegetation indices.

**Table 1:** Information about the MS features.

Features	Formulas	References
Normalized difference vegetation index	NDVI=(NIR−R)/(NIR+R)	(Li Z. et al., 2023)
Normalized Difference Red-Edge	NDRE=(NIR−RE)/(NIR+RE)	(Li Z. et al., 2023)
Green NDVI	GNDVI=(NIR−G)/(NIR+G)	(Hancock and Dougherty, 2007)
Canopy chlorophyll content index	$\begin{array}{l} CCCI=(NIR-RE)/(NIR+RE)/\\ \left(NIR-R)/(NIR+R\right) \end{array}$	(Li Z. et al., 2023)
Green ratio vegetation index	GRVI=NIR/G	(Nguyen et al., 2023)
Red-Green Ratio	RGR=R/G	(Gamon and Surfus, 1999)
RedEdge Ratio Index 1	RRI1=NIR/RE	(Ehammer et al., 2010)
RedEdge Ratio Index 2	RRI2=RE/R	(Ehammer et al., 2010)
Adjusted transformed soil-adjusted Vegetation Index	$\begin{array}{l} ATSAVI=1.22(NIR-1.22R-1.22)/\\ (1.22NIR+R-1.22*0.03+0.08*\\ \left(1+1.22^{2})\right) \end{array}$	(He et al., 2007)
Chlorophyll Index Green	CIg=(NIR/G)−1	(Jr. Hunt et al., 2011)
Chlorophyll IndexRedEdge	CIre=(NIR/RE)−1	(Jr. Hunt et al., 2011)
Ideal vegetation index	IVI=(NIR−0.03)/(1.22R)	(Main et al., 2011)
Difference vegetation index	DVI=NIR−R	(Tucker, 1979)
Weighted Difference Vegetation Index	WDVI=NIR−1.22R	(Ehammer et al., 2010)
Transformed Vegetation Index	$TVI=\sqrt{NDVI+0.5}$	(Tran et al., 2022)
Wide Dynamic Range Vegetation Index	WDRVI=(0.1NIR−R)/(0.1NIR+R)	(Ahamed et al., 2011)
Transformed NDVI	$TNDVI=\sqrt{\left(NIR-R)/(NIR+R+0.5\right)}$	(LUO et al., 2006)
Soil-adjusted vegetation index	SAVI=1.5×(NIR−R)/(NIR+R+0.16)	(Li Z. et al., 2023)
Green difference vegetation index	GDVI=NIR−G	(Tucker et al., 1979)
Green soil adjusted vegetation index	GSAVI=1.5×(NIR−G)/(NIR+G+0.5)	(Da Luz et al., 2022)
Norm G	NormG=G/(NIR+R+G)	(Abdollahi and Zakeri, 2022)
Norm NIR	NormNIR=NIR/(R+G+NIR)	(Abdollahi and Zakeri, 2022)
Norm R	NormR=R/(R+G+NIR)	(Abdollahi and Zakeri, 2022)
Normalized green red difference index	NGRDI=(G−R)/(G+R)	(Ahamed et al., 2011)
Redness Index	RI=(R−G)/(R+G)	(Tran et al., 2022)
Chlorophyll vegetation index	$CVI=NIR(R/G^{2})$	(Datt et al., 2003)
Ratio Vegetation Index	RVI=NIR/R	(Haboudane et al., 2004)
Nonlinear Vegetation Index	NLI=(0.12NIR−R)/(0.12NIR+R)	(Roujean and Breon, 1995)
Modified Nonlinear Vegetation Index	$\begin{array}{l} MNLI=(1.5NIR^{2}-1.5G)/\\ \left(NIR^{2}+R+0.5\right) \end{array}$	(Jordan, 1969)
Optimized Soil-Adjusted Vegetation Index	OSAVI=(NIR−R)/(NIR+R+0.16)	(Ihuoma and Madramootoo, 2019)
Transformed Chlorophyll Absorption Ratio Index	$\begin{array}{l} TCARI=3((RE-R)-\\ 0.2(RE-G)(RE/R)) \end{array}$	(Bagheri, 2020)
Modified Chlorophyll Absorption Ratio Index	$\begin{array}{l} MCARI=((RE-R)-\\ 0.2(RE-G))(RE/R) \end{array}$	(Li et al., 2019)
Green Chlorophyll Index	GCI=(NIR/G)−1	(Raper and Varco, 2015)
Red Edge Chlorophyll Index	RECI=(NIR/RE)−1	(Jiang et al., 2008)
Modified Ratio Vegetation Index	MRVI=(NIR/R−1)/(NIR/R+1)	(Baret and Guyot, 1991)

### 2.5 Modeling framework

In this section, we provide an overview of our proposed modeling framework, as depicted in **Figure 2**. The set of 35 yield-sensitive vegetation indices, constructed within this study, may potentially contain redundant features. Utilizing all these indices as direct input features can be detrimental to the performance of our yield prediction model. Consequently, a data pre-processing approach is imperative to select and refine the features within the dataset. Feature selection methods include filtering, embedding and packing methods. Our proposed modeling framework primarily consists of two key steps. First, we employ an integrated feature selection method that leverages the PC and RF importance to filter and screen the vegetation indices, ultimately identifying the features crucial for yield prediction. Subsequently, based on the refined feature subset obtained through feature selection, we proceed with recursive feature elimination to further optimize the feature subset. Upon the completion of feature selection, both the full feature subset and the subsets obtained through various feature selection methods are used as input features for both the Cubist and RNN models. To assess model performance, the feature subset is randomly divided using the 10-fold cross-validation method. In this approach, each of the 10 folds serves as the validation set, while the remaining 9 folds constitute the training set for ten iterations. The results obtained across these iterations are consolidated, and the mean value is considered as the final accuracy measure for the model.

#### 2.5.1 Pearson correlation coefficient and random forest importance

PC falls under the category of filtered feature selection methods. It stands as one of the most commonly employed statistical tools for quantifying the relationships between linearly correlated variables. This coefficient provides a numerical measure of the strength of the relationship between variables. In the context of feature selection, the PC proves valuable in assessing the linear correlation between each feature and the target variable. This information aids in the selection of highly correlated features with the target variable, thereby enhancing the performance and explanatory capacity of the model (Zhang et al., 2020). Its formula is as follows:


Eq.1 $$r_{\left(X,Y\right)}=\frac{Cov(X,Y)}{\sqrt{Var(X)Var(Y)}}$$

Where *Cov* (*X*, *Y*) represents the covariance of *X* and, *Y VAR*(*X*) is the variance of *X*, and *VAR*(*X*) is the variance of *Y*.

The RF is a potent machine learning algorithm widely used for feature selection and predictive tasks. By integrating multiple decision trees (Chen et al., 2022), it enables more accurate and robust predictions. In a RF, feature importance is a crucial concept used to assess each feature’s contribution to the model’s predictive performance. This model is composed of several decision trees, each constructed using random subsets of data and features, which enhances generalization and mitigates overfitting.To quantify a feature’s importance, the model computes the difference in prediction error when the feature is included versus when it is excluded in all decision trees. These differences are averaged to determine the feature’s importance. Features with higher importance values play a more significant role in the model’s predictive power. These importance scores offer insights into which features have a greater impact on the target variable, aiding in feature selection and model interpretation. The RF is a comprehensive model that combines various decision trees, each constructed using bagging, and calculates the cumulative importance score for each feature based on their impurity reduction values across all the trees. This score provides an overall measure of a feature’s impact on the model’s predictive performance. We utilized R (version 4.3) for computing the importance scores in the Random Forest model.

#### 2.5.2 Integrated feature selection based on Pearson correlation coefficient and random forest importance

The integrated feature selection method, which combines the PC and RF importance, constitutes the first step of our proposed feature selection approach. In this initial phase, we exclusively utilize the 35 vegetation index features spanning the five bands of the multispectral data to ensure the retention of crucial spectral information. Subsequently, we calculate the importance of each feature independently using both the PC and RF techniques. The importance scores are then ranked and visualized, facilitating the selection of a threshold that distinguishes obviously less important features from others. Features with importance scores exceeding the chosen threshold are retained, while those falling below are eliminated. We posit that there may be significant features present within the subset selected through both the PC and RF metrics. Hence, we use their union as a foundation for further feature optimization.

The second step in our integrated feature selection method involves the RFE technique. RFE, a wrapper-type feature selection method, progressively identifies the features that have the most substantial impact on model performance by iteratively removing less important features (Li Y. et al., 2023). This method operates by constructing a model and selecting and eliminating features based on their importance within the model, continuing until a predetermined number of features or a stopping condition is met. In our approach, we employ the subset of union features obtained from the PC and RF importance methods as input features for RFE. The RFE method utilizes all available features to train the base model, which encompasses both the Cubist and RNN models. Within this base model, the importance of each feature is assessed, and less important features are systematically eliminated through iterative steps. This process culminates in the identification of the subset of features that most effectively enhance the performance of both the Cubist and RNN models.

#### 2.5.3 Cubist model

Cubist model is an integrated learning method that combines the advantages of decision tree. It serves as a versatile machine learning model designed for regression tasks and excels at capturing complex non-linear relationships. This is achieved through the fusion of regression trees and the identification of interactions between features, making it less susceptible to the influence of outlier noise. The Cubist model often demonstrates robust performance (Ou et al., 2019). In addition, the Cubist model requires less scaling and normalisation of the data. This can save time and effort in data pre-processing in practical applications. The basic principle of Cubist is to first divide the dataset into regions based on similarity and then use ordinary least squares regression to predict the variables of interest within each region. The Cubist model is as follows:


$$\begin{array}{l} \text{If [condition is true]}\\ \text{ then [regress]}\\ \text{else [apply next rule]} \end{array}$$

The continuous and categorical variables can be used in conditions, but only continuous variables can be used in regression equations (Han et al., 2023).

#### 2.5.4 RNN model

**Figure 3:**
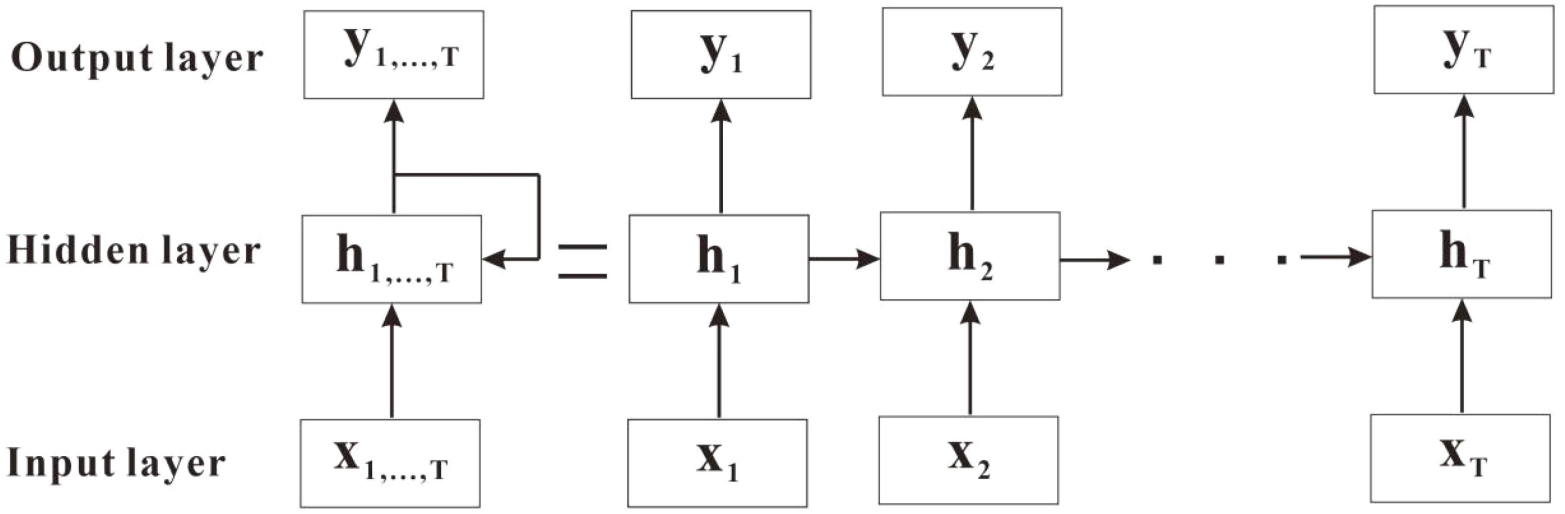
RNN structure.

RNN is a deep learning model well-suited for handling sequential data. Its standout feature lies in its inherent memory, allowing it to consider prior information, which influences present predictions or outputs. RNN is typically trained via backpropagation algorithms. RNN is composed of three key components: the input layer, the hidden layer, and the output layer, with its essential cyclic structure facilitating the transfer of information across different time steps (Murata et al., 2023). The structure of RNN is depicted in **Figure 3**.

### 2.6 Model accuracy assessment

To gauge the predictive performance of our model, we have chosen two key parameters for evaluation: R-squared (R^2^) and Root Mean Square Error (RMSE). A higher R^2^ value, closer to 1, signifies a better fit of the model to the data. Conversely, a lower RMSE value, closer to 0, indicates greater accuracy in the model’s predictions. In essence, these parameters provide essential metrics for assessing the model’s effectiveness. Conversely, if the R^2^ is low and the RMSE is high, it implies that the model lacks accuracy and fails to deliver the anticipated predictive power. The formulas for these parameters are as follows:


Eq. 2 $$R^{2}=1-\frac{\sum_{i=1}^{n}{\left({\overset{⌢}{y}}_{i}-y_{i}\right)}^{2}}{\sum_{i=1}^{n}{\left(y_{i}-\bar{y}\right)}^{2}}$$

Eq. 3 $$RMSE=\sqrt{\frac{\sum_{i=1}^{n}{\left({\hat{y}}_{i}-y_{i}\right)}^{2}}{N}}$$

where ${\text{y}}_{\text{i}}$ is the observed value, is the predicted value, where $\hat{{\text{y}}_{\text{i}}}$ is the predicted value, is the mean of the observed values, and N is the sample size. $\bar{\text{y}}$ is the mean of the observed values, and *N* is the sample size.

## 3 Results

### 3.1 Descriptive statistics

**Figure 4:**
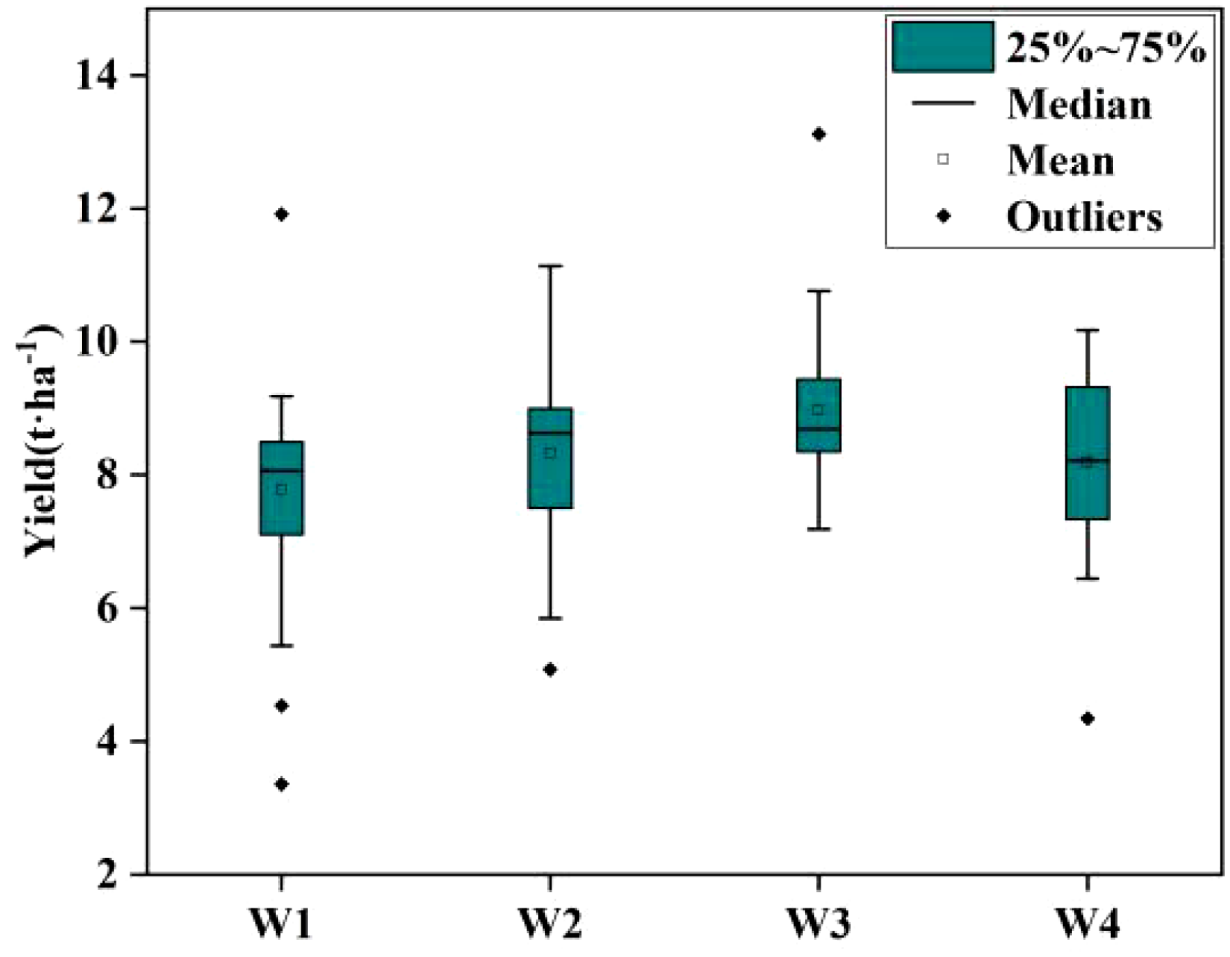
Observed yield distribution in 2023.

**Figure 4** and **Table 2** present the descriptive statistics for wheat yield across 120 plots subjected to four different treatments. The average yield across all sampled plots in this experiment stood at 8.31 t·ha^-1^. It’s noteworthy that wheat yield exhibited variations among the three extreme climate treatments and the treatments with sufficient water. Treatment W3 boasted the highest average wheat yield at 8.97 t·ha^-1^, whereas treatment W1 had the lowest average yield at 7.77 t·ha^-1^. Interestingly, the mean yield under treatment W4 was slightly lower than that of treatment W2, but significantly higher than the yield in treatment W1. This observation suggests that, in this experiment, the extreme drought treatment (W2) had a more pronounced impact on yield compared to the extreme rainfall treatment (W4). Analyzing the range, standard deviation (SD), quantitative statistics, and coefficient of variation (CV) for all plots and plots under each water treatment reveals significant differences in yields among the various experimental treatments, with distinct data separation.

**Table 2:** Descriptive statistics of observed yield(t·ha^-1^).

Category	Observations	Min	Max.	SD	Q25	Q50	Q75	Mean	CV
All datasets	120	3.35	13.11	1.40	7.69	8.43	8.99	8.31	16.9%
W1 dataset	30	3.35	11.91	1.56	7.12	8.06	8.48	7.77	20%
W2 dataset	30	5.08	11.13	1.39	7.60	8.62	8.98	8.32	16.7%
W3 dataset	30	7.18	13.11	1.19	8.36	8.68	9.40	8.97	13.3%
W4 dataset	30	4.34	10.17	1.26	7.34	8.21	9.29	8.19	15.4%

The yield data for ten wheat cultivars under varying water treatments is presented in **Table 3**. We observed that the highest yields were achieved in the W3 treatment, with the exception of Denghai202, Bainong207, Jimai22, and Shannong36. Denghai202 performed best in the W1 treatment, while Shannong36 excelled in the W2 treatment. These results indicate that Denghai202 and Shannong36 exhibit notable drought tolerance. Additionally, Bainong207 and Jimai22 produced the highest yields in the W4 treatment, suggesting their high water tolerance.

**Table 3:** Yield data for ten wheat cultivars across four different water treatment conditions(t·ha^-1^).

Treatment	Denghai202	Bainong207	Jimai22	Shannong36	Letu808	Dunmai88	Yannong1212	Bainong58	Zhongmai578	Luyuan502
W1	9.78	8.14	8.36	7.86	7.07	7.35	5.00	8.09	8.41	8.23
W2	8.45	7.55	8.11	9.13	8.70	9.07	7.17	8.36	7.06	9.58
W3	8.26	8.56	8.78	8.63	8.71	10.70	8.86	8.45	8.87	8.85
W4	8.12	9.39	9.04	7.46	8.49	9.40	7.11	7.90	7.90	8.71

### 3.2 Feature screening combination

**Figure 5:**
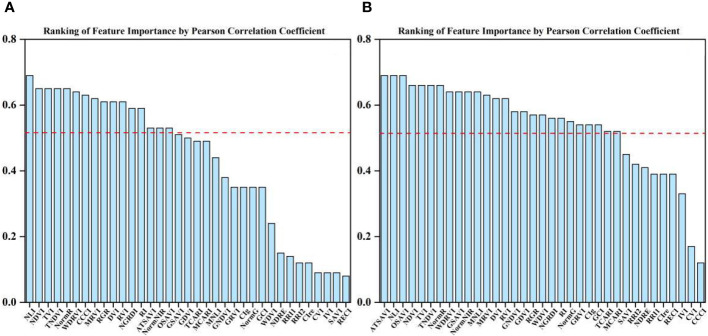
Ranking of importance of PC features at flowering and grain filling stages. **(A)** flowering stage, **(B)** grain filling stage.

**Figure 6:**
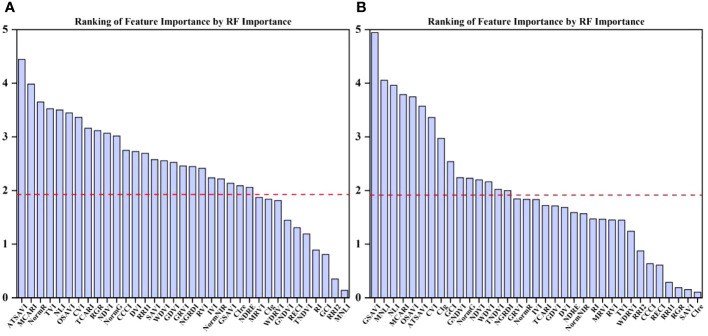
Ranking of importance of RF importance features at flowering and grain filling stages. **(A)** flowering stage, **(B)** grain filling stage.

**Figure 7:**
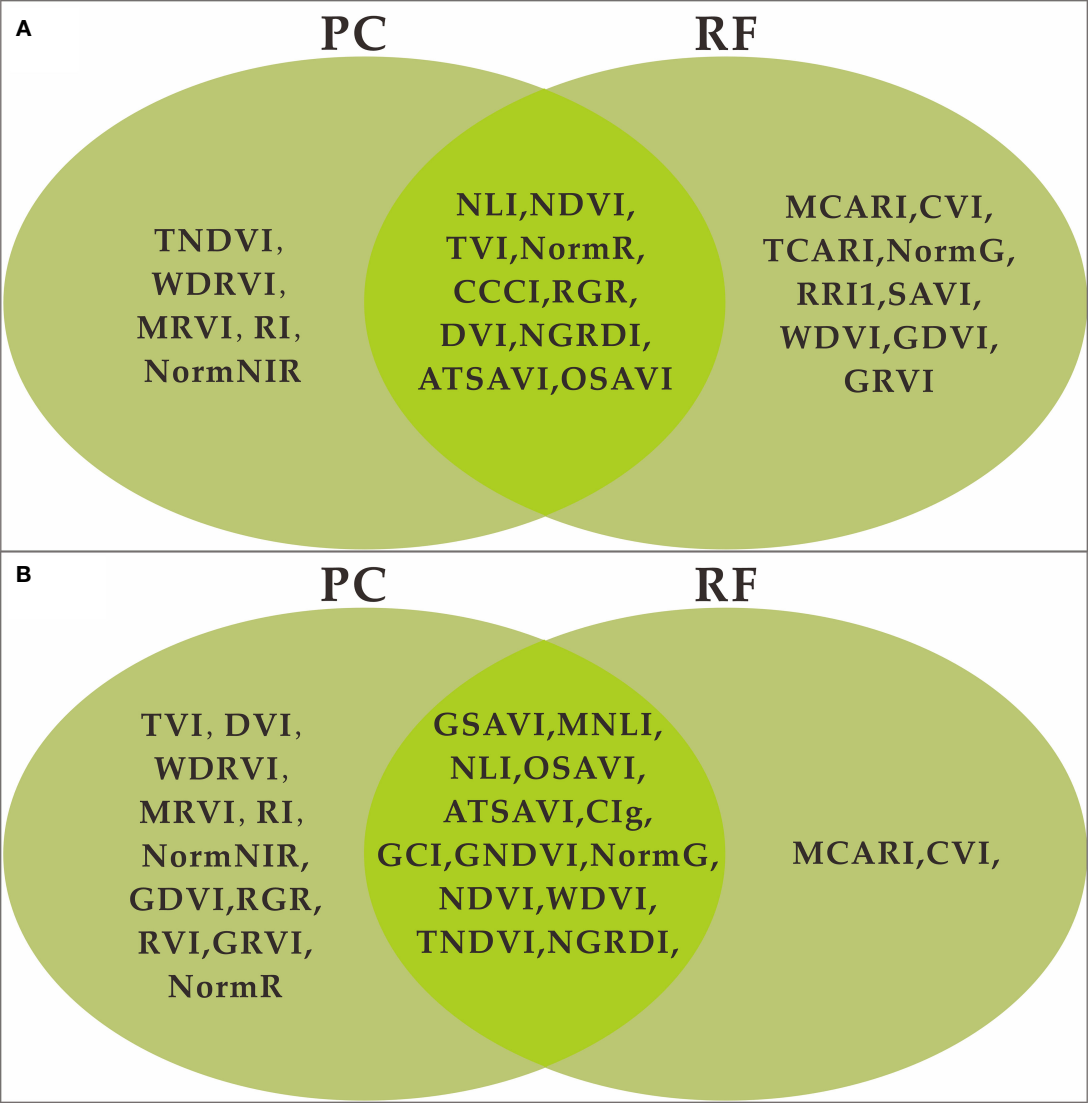
Feature union set of PC and RF feature selection. **(A)** flowering stage, **(B)** grain filling stage.

PC and RF importance were employed to rank the importance of vegetation indices at the two fertility stages. The rankings indicated that the NLI was the most important feature during the flowering stage, while the ATSAVI, NLI, and OSAVI were the most significant during the grain filling stage according to the PC ranking. In contrast, the RF importance ranking identified ATSAVI as the primary feature during the flowering stage and GSAVI as the most important during the grain filling stage. However, there were some features with low importance rankings in both the PC and RF importance, which could potentially impact the model’s performance negatively. The significance of the correlation between vegetation indices and yield was also considered (**Table 4**). To mitigate the potential impact of these less important features on the prediction accuracy of the model, we introduced thresholds of 0.53 and 1.9 for the two feature selection methods to filter the importance of features. As demonstrated in **Figures 5** and **6**, this approach retained features with importance coefficients exceeding 0.53 in the PC ranking and features with coefficients exceeding 1.9 in the RF importance ranking. Features that fell below these thresholds were removed from consideration, resulting in the creation of four feature subsets. During the flowering stage, the PC method retained 16 features, while the RF importance method retained 20 features. The intersection of these methods, as seen in **Figure 7A**, identified 10 common features. The union of features from both methods totaled 25 features. Similarly, during the grain filling stage, the PC method retained 24 features, and the RF importance method retained 15 features. The intersection of these methods, as shown in **Figure 7B**, revealed 13 common features. The union of these features resulted in a total of 26. This comprehensive feature subset obtained by combining results from both fertility stages serves as a foundation for the subsequent step of feature selection and refinement.

**Table 4:** Statistical significance testing of features (*p*-value).

Features	Flowering	Grain filling
NLI	***	***
NDVI	***	***
TVI	***	***
TNDVI	***	***
NormR	***	***
WDRVI	***	***
CCCI	***	***
MRVI	***	***
RGR	***	***
DVI	***	***
RVI	***	***
NGRDI	***	***
RI	***	***
ATSAVI	***	***
NormNIR	***	***
OSAVI	***	***
GSAVI	***	***
GDVI	***	***
TCARI	***	***
MCARI	***	***
MNLI	***	***
GNDVI	***	***
GRVI	**	***
CIg	**	***
NormG	**	***
GCI	**	***
WDVI	*	***
CVI	/	***
IVI	/	***
NDRE	/	***
RRI1	/	***
RRI2	/	***
CIre	/	**
SAVI	/	/
RECI	/	/

* respresents p<0.05, ** represents p<0.01, *** represents p<0.001,/represents no significant.

In the PCRF integration step, we obtained two feature subsets containing 25 and 26 features for the vegetation indices during the flowering and grain filling stages, respectively. Subsequently, we performed wrapper-based RFE feature selection on each of these two feature subsets individually. The feature selection was carried out using the same random seeds in the experiment and a patience parameter of 10. In other words, the RFE process was terminated if there was no improvement in performance over a cumulative span of 10 iterations. Following the feature selection process, we had subsets of features for both the Cubist and RNN models. The subset derived from the flowering stage contained 13 features for Cubist and 11 features for RNN. Meanwhile, the subset obtained during the grain filling stage comprised 15 features for Cubist and 18 features for RNN (**Table 5**). These feature sets were utilized for the final construction of the yield prediction model.

**Table 5:** Number of features included in the subset.

Stage	Number					
	PC	RF	PC&RF Uni.	PC&RF Inter.	PCRF-RFE(Cubist)	PCRF-RFE(RNN)
Flowering	16	20	25	10	13	11
Grain filling	24	15	26	13	15	18

### 3.3 Model validation analysis

**Figure 8:**
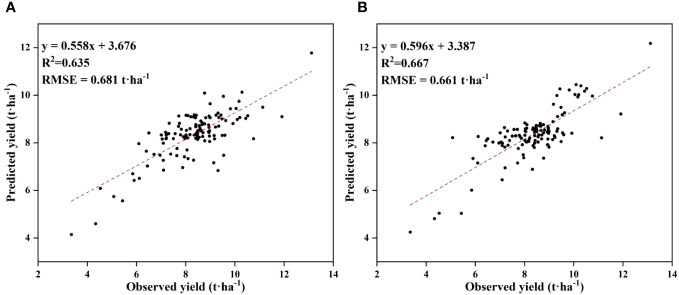
Distribution of observed and predicted yields for the optimal yield prediction model during the flowering and grain filling stages. **(A)** flowering stage, **(B)** grain filling stage.

**Figure 9:**
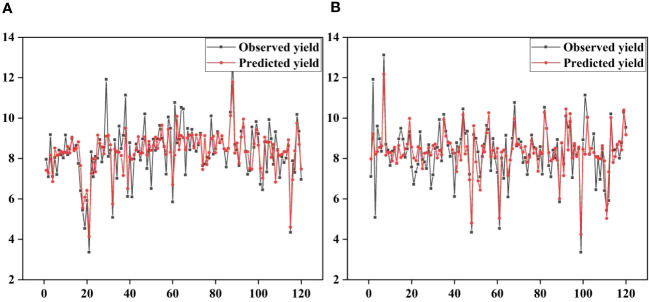
Observed and predicted yields for the optimal yield prediction model during the flowering and grain filling stages. **(A)** flowering stage, **(B)** grain filling stage.

Cubist and RNN yield prediction models were constructed based on all the features at the flowering and filling stages, the feature subsets selected by the PC and the RF importance method, and the feature subsets selected by the PCRF-RFE method proposed in this study, respectively. The accuracies of the prediction models are shown in **Table 6**. The accuracy of the Cubist yield prediction model constructed based on the feature subset selected by the PCRF-RFE method at the flowering stage was the best (R^2^ = 0.635, RMSE = 0.681 t·ha^-1^) (**Figure 8A**). This was followed by the accuracy of the RNN yield prediction model constructed based on the feature subset obtained by the PCRF-RFE method, with an R^2^ of 0.607, slightly higher than that of the Cubist model constructed based on the feature subset obtained by the PC method. During the grain filling stage, the Cubist yield prediction model constructed based on the subset of features selected by the PCRF-RFE method had the best performance (R^2^ = 0.667, RMSE = 0.661 t·ha^-1^) (**Figure 8B**). In contrast, the RNN yield prediction model constructed based on all features had the worst accuracy, with an R^2^ of 0.512. The Cubist and RNN yield prediction models constructed based on the subset of features obtained by the same feature selection method at the grain filling stage had higher accuracy than those at the flowering stage. The performance of the Cubist and RNN prediction models could be significantly improved by using the feature selection method. When using the intersection or union of the feature subsets obtained by the PC and RF importance methods as the feature subsets to construct the Cubist and RNN yield prediction models, the accuracy of the model was also greatly improved compared to that of all the features used as input variables. The R^2^ could reach 0.606 at the highest, but it was still lower than that of the yield prediction model constructed based on the feature subsets selected by the PC or RF importance methods. A comparative analysis between the observed and predicted yields from the Cubist model, conducted at both the flowering and grain filling stages, demonstrated that the grain filling stage exhibited greater consistency with the observed yields and outperformed the flowering stage (**Figure 9**).

**Table 6:** Model accuracy of different feature selection methods.

Models	Subsets	Flowering		Grain Filling	
		R^2^	RMSE/(t·ha^-1^)	R^2^	RMSE/(t·ha^-1^)
Cubist	All Features	0.509	0.795	0.541	0.782
	PC	0.601	0.801	0.641	0.73
	RF	0.586	0.772	0.614	0.787
	PC&RF Uni.	0.596	0.685	0.602	0.736
	PC&RF Inter.	0.580	0.797	0.606	0.656
	PCRF-RFE	0.635	0.681	0.667	0.661
RNN	All Features	0.492	0.893	0.512	0.961
	PC	0.578	0.829	0.598	0.788
	RF	0.561	0.825	0.587	0.883
	PC&RF Uni.	0.555	0.961	0.571	1.020
	PC&RF Inter.	0.552	0.819	0.562	1.091
	PCRF-RFE	0.607	0.793	0.628	0.872

**Figure 10:**
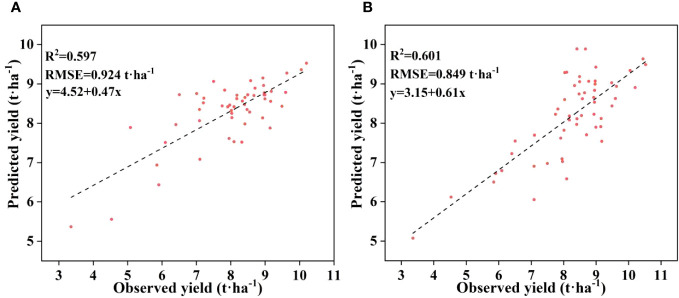
Distribution of observed and predicted yields for the Cubist yield prediction model. **(A)** flowering stage, **(B)** grain filling stage.

To assess the adaptability of our integrated feature selection method in diverse environments and the applicability of our predictive model, we have chosen the best-performing models from two distinct fertility stages (**Figure 10**). We intend to construct a Cubist model using the optimal variable combination derived from the PCRF-RFE integrated feature selection method. Specifically, we will use data from the W1 and W2 treatments as the training set, and data from the W3 and W4 treatments as the validation set. The accuracy of the Cubist model consistently demonstrates strong performance, with R^2^ exceeding 0.59. Furthermore, the lowest RMSE achieved is as low as 0.849 t·ha^-1^. Notably, the model’s accuracy at the grain filling stage surpasses that at the flowering stage. In summary, the PCRF-RFE feature selection method exhibits its superiority by consistently delivering high accuracy, even when dealing with data from distinct environmental settings for training and validation. This also underscores the robust adaptability of the Cubist model in various scenarios.

### 3.4 The results of best prediction models

**Figure 11:**
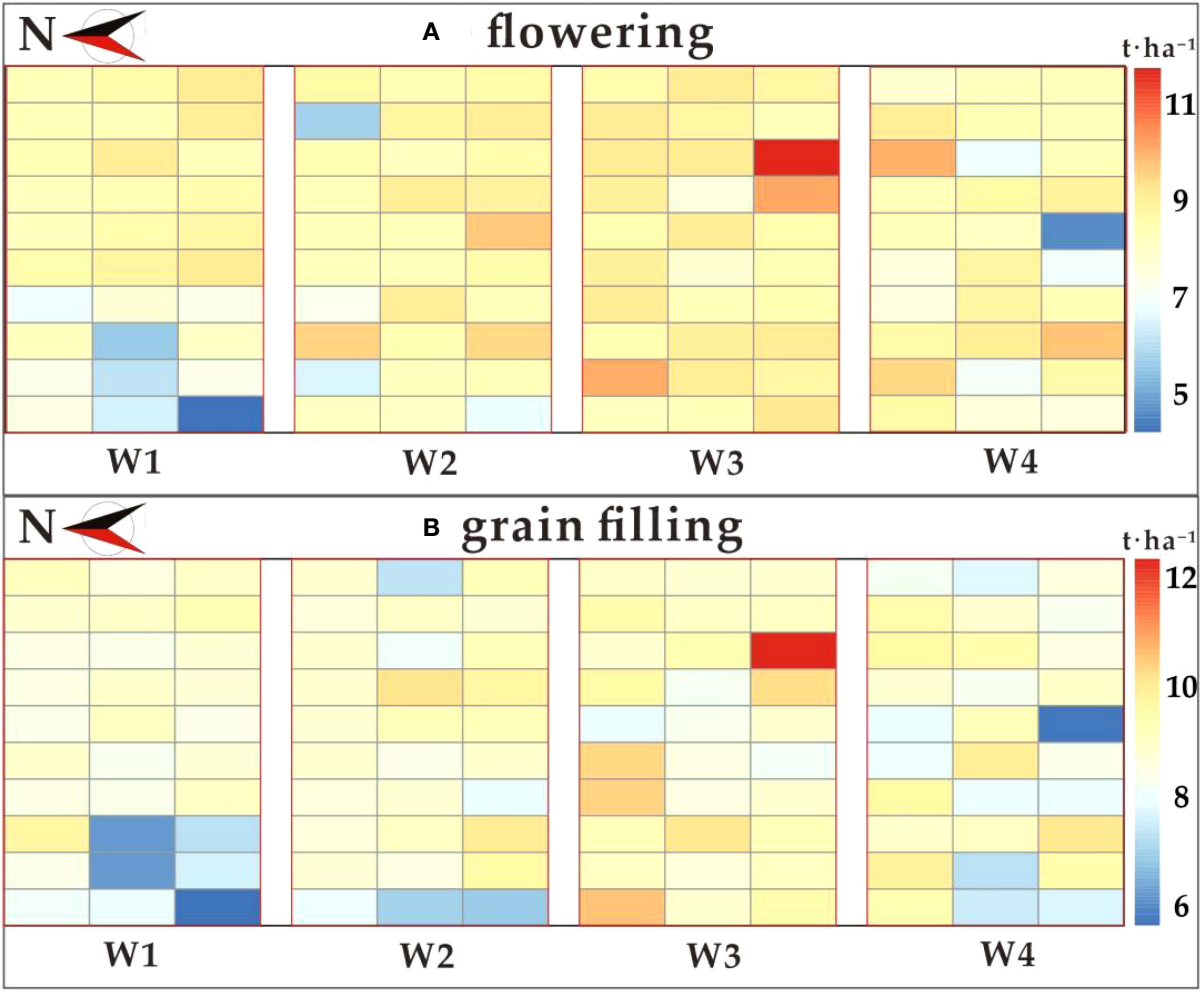
Distribution of predicted yields from the optimal yield prediction model. **(A)** flowering stage; **(B)** grain filling stage.

In this study, a diverse set of vegetation indices was selected at both the flowering and grain filling stages. The yield prediction accuracy of the Cubist model, constructed using our developed PCRF-RFE method with different feature subsets as input variables, showed the best performance at both the flowering and grain filling stages. These models were used to generate predicted yield distributions (**Figure 11**). The results of the t-test distribution of yield between treatments at the flowering stage are presented in **Table 7**. The *p*-values between W1 and W3, W2 and W3, and W4 and W3 were all less than 0.01, indicating highly significant differences among these treatments. Furthermore, the *p*-values between W1 and W4, and W1 and W2 were less than 0.05, signifying significant differences between these treatment pairs. However, the *p*-values between W2 and W4 were greater than 0.05, suggesting that there was no significant difference between the effects of W2 and W4 on wheat yield. This observation indicates that the W2 and W4 had similar effects on wheat yield. Moreover, the *p*-value between W1 and W2 was significantly smaller than that between W1 and W4, indicating a greater difference between W2 and W1. The yield ranking between treatments was found to be W3 > W2 > W4 > W1. The results of the t-test distribution of yield between different treatments at the filling stage are shown in **Table 8**, and the significance of the treatments was similar to that at the flowering stage. The yield ranking between treatments remained W3 > W2 > W4 > W1.Based on the observed yield results, W3 had the highest yield between 7.184-13.117 t·ha^-1^, followed by W2 and W4 treatments, with the lowest observed yield in W1. These results align with the predicted yield distribution of the Cubist model, constructed using the PCRF-RFE method to select feature subsets at the flowering and grain filling stages. The consistent predicted yield distribution further affirms the utility of the feature selection method proposed in this study for winter wheat yield prediction.

**Table 7:** Results of *t*-test for flowering stage.

Feature	*t*	*p*-Value
W1 vs W2	54.457	0.04
W1 vs W3	52.423	0.000
W1 vs W4	57.664	0.13
W2 vs W3	57.653	0.009
W2 vs W4	56.134	0.646
W3 vs W4	54.426	0.005

**Table 8:** Results of *t*-test for grain filling stage.

Feature	*t*	*p*-Value
W1 vs W2	48.693	0.041
W1 vs W3	56.716	0.000
W1 vs W4	53.703	0.044
W2 vs W3	53.007	0.002
W2 vs W4	56.255	0.916
W3 vs W4	56.912	0.005

## 4 Discussion

Their importance for yield prediction was ranked during both the flowering and grain filling stages using the PC and RF importance methods. To enhance the model’s robustness and reduce complexity, we applied thresholds of 0.53 and 1.9 to filter out low-importance features. This approach aligns with a previous study (Marques Ramos et al., 2020) and proved effective in feature selection. We introduced a novel step in the form of PCRF integration to select features from subsets obtained via PC and RF importance methods. This integrated approach minimizes potential bias resulting from a single selection method. Subsequently, we utilized the wrapper-based RFE feature selection technique to further refine the model’s input features, enhancing predictive performance. Our integrated feature selection method encompasses three distinct types: filtering, wrapping, and embedding, thereby capitalizing on the strengths of each. The PC method, as a filtering approach (Pocas et al., 2015), efficiently assesses linear relationships between features and target variables, making it computationally fast. However, its performance may be limited when dealing with nonlinear relationships. In contrast, the RF importance method, classified as an embedding method (Ma et al., 2018), excels at capturing nonlinear feature-target interactions, exhibits greater resilience to outliers and noise, and enhances model robustness. The RFE method (Koc et al., 2022), a wrapper technique, excels at modeling intricate feature-target relationships, offers high interpretability, and can identify feature interactions. Therefore, we employed the RFE method as the second step in our integrated feature selection approach.

**Figure 12:**
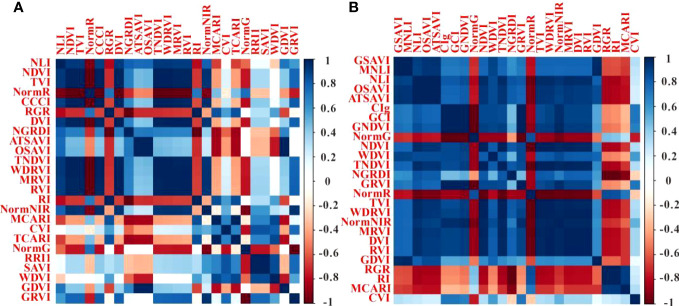
Correlation matrix between sensitive features. **(A)** represents flowering stage, **(B)** represents grain filling stage.

We have considered the union of feature subsets identified by both PC and RF Importance methods as the set of features most sensitive to yield. The correlation between these sensitive features is showed in **Figure 12**. During the flowering stage, a strong correlation is observed among NDVI, TVI, CCCI, DVI, TNDVI, WDRVI, MRVI, RVI, and NormNIR. Notably, the feature subset obtained through the PC method encompasses more features exhibiting robust autocorrelation, leading to a more accurate model construction. However, it is worth mentioning that this may be partly due to potential overfitting resulting from the data screening process employed by the PC method. Conversely, the RF Importance method can simultaneously consider multiple features, effectively capturing non-linear relationships between the features and the target variable (AlSagri and Ykhlef, 2020), thus improving overall model performance. Despite the PC feature subset comprising 16 features at this point, a significant portion of them displays strong autocorrelation, rendering the RF Importance approach more reliable in terms of performance. During the filling period, a substantial number of sensitive features exhibit autocorrelation. As a result, the feature subset derived from the RF Importance method contains only 15 features, while the PC method achieves higher predictive accuracy due to the inclusion of more autocorrelated features. Although the intersection of the PC and RF Importance methods yields a feature subset with the fewest number of features, containing numerous autocorrelated features, it remains a challenge to achieve a significantly improved prediction accuracy.

In our study, we conducted a comprehensive comparison of Cubist and RNN yield prediction models using various feature subsets. The results consistently demonstrated that models constructed with feature subsets obtained from the PCRF integration step outperformed other methods in terms of accuracy for both the flowering and grain filling stages. This underscores the effectiveness of the PCRF integrated feature selection method in optimizing feature selection and model construction, which is in line with a previous study (Yin et al., 2023). We systematically compared and contrasted the Pearson correlation coefficient method, the random forest importance selection method, and the PCRF-RFE integrated feature selection method in yield prediction models at both flowering and filling stages. The results revealed that the constructed yield prediction models, whether based on the PCRF-RFE method or on a single feature selection method, consistently achieved higher accuracy compared to models using all features. This underscores the substantial improvement brought about by feature selection methods on model performance and reaffirms the critical role of feature selection in enabling models to better capture yield-related features and enhance prediction accuracy (Kohavi and John, 1997; Jeon and Oh, 2020; Yin et al., 2023). We examined the performance of models using feature subsets obtained from different feature selection methods. During both the flowering and grain filling stages, the model constructed using the feature subset derived from the PCRF-RFE method demonstrated superior performance. This underscores the effectiveness of our proposed integrated feature selection method and its capacity to enhance the accuracy of prediction models across different fertility stages. Additionally, we explored the construction of predictive models using the intersection or union of feature subsets obtained from the PC method and the RF importance selection method as input variables. The results indicated that the use of intersection or union as feature subsets could also significantly improve model performance, although the accuracy was slightly lower compared to models based on a single feature selection method.

The Cubist model employed in this study is an integrated machine learning algorithm designed for regression tasks. It leverages regression trees to efficiently capture feature relationships by considering multiple features simultaneously during node splits. This characteristic is particularly advantageous when used in conjunction with RFE methods (Zhang et al., 2023). Moreover, the Cubist model is relatively less affected by outliers and noise, rendering it more robust when applied to real-world data. Within the RFE process, this robustness helps mitigate the impact of outliers, resulting in a more accurate subset of features (Xiao et al., 2022; Zhang et al., 2023). Using the Cubist model as the base for the RFE method enhances its ability to discern the relationship between the model itself and the subset of features through iterative training and feature selection. This iterative process increases the likelihood of identifying an optimal subset of features. These findings align with the conclusions drawn in a previous study (Zhou et al., 2022), which highlighted that the combination of the Cubist model and the RFE method yielded the highest accuracy and the lowest uncertainty. This validates our choice of the RFE method and is consistent with the results of a previous study (Xiao et al., 2022).

Our research offers valuable insights in the field of crop yield prediction, particularly in the development and implementation of integrated feature selection methods. Our study not only enhances our comprehension of the significance of feature selection methods in crop yield prediction but also provides practical guidance for agricultural applications. Furthermore, there is potential for exploring additional feature selection techniques and model combinations, as well as their application to diverse crops and environmental conditions in future research.

## 5 Conclusion

In this study, we utilized a UAV remote sensing platform to capture multispectral images during both the flowering and grain-filling stages of winter wheat. After processing these images, we calculated 35 vegetation indices known for their sensitivity to crop yield. Subsequently, we applied the Pearson correlation coefficient and random forest importance methods to select the most relevant indices among the 35. We then derived feature subsets through these two feature selection methods and combined them to create the input feature set for the RFE method. We used Cubist and RNN models as base models to identify the most optimal feature subsets for each growth stage. The results were clear: the Cubist yield prediction model, based on feature subsets obtained through the PCRF-RFE feature selection method, demonstrated remarkable performance in both the flowering stage (R^2^ = 0.635, RMSE = 0.681) and the grain filling stage (R^2^ = 0.667, RMSE = 0.661). Notably, the model’s accuracy was consistently higher during the grain-filling stage compared to the flowering stage. These findings provide compelling evidence supporting the practicality and viability of our proposed PCRF-RFE method, offering valuable insights for future research in the field of yield prediction.

## Data availability statement

The data analyzed in this study is subject to the following licenses/restrictions: Data set design project confidentiality. Requests to access these datasets should be directed to lizongpeng1996@163.com.

## Author contributions

ZL: Conceptualization, Data curation, Formal analysis, Methodology, Software, Validation, Writing – original draft, Writing – review & editing. XZ: Funding acquisition, Supervision, Validation, Writing – review & editing. QC: Data curation, Formal Analysis, Methodology, Writing – review & editing. ZC: Conceptualization, Funding acquisition, Writing – review & editing. WZ: Data curation, Project administration, Software, Writing – original draft. YL: Data curation, Formal analysis, Writing – original draft. BM: Data curation, Writing – original draft.

## References

[B1] Abdel-Rahman E. M. Ahmed F. B. Ismail R. (2013). Random forest regression and spectral band selection for estimating sugarcane leaf nitrogen concentration using eo-1 hyperion hyperspectral data. Int. J. Remote Sens. 34, 712–728. doi: 10.1080/01431161.2012.713142

[B2] Abdollahi A. Zakeri N. (2022). Cospectrality of multipartite graphs. Ars Mathematica Contemporanea 22, 1. doi: 10.26493/1855-3974.2332.749

[B3] Ahamed T. Tian L. Zhang Y. Ting K. C. (2011). A review of remote sensing methods for biomass feedstock production. Biomass Bioenergy 35, 2455–2469. doi: 10.1016/j.biombioe.2011.02.028

[B4] AlSagri H. Ykhlef M. (2020). Quantifying feature importance for detecting depression using random forest. Int. J. Advanced Comput. Sci. Appl. 11, 628–635. doi: 10.14569/IJACSA.2020.0110577

[B5] Azizi K. Ayoubi S. Nabiollahi K. Garosi Y. Gislum R. (2022). Predicting heavy metal contents by applying machine learning approaches and environmental covariates in west of iran. J Geochem Exploration 233, 106921. doi: 10.1016/j.gexplo.2021.106921

[B6] Bagheri N. (2020). Application of aerial remote sensing technology for detection of fire blight infected pear trees. Comput. Electron. Agric. 168, 105147. doi: 10.1016/j.compag.2019.105147

[B7] Bai Z. Xie M. Hu B. Luo D. Wan C. Peng J.. (2022). Estimation of soil organic carbon using vis-nir spectral data and spectral feature bands selection in Southern Xinjiang, China. Sensors 22, 6124. doi: 10.3390/s22166124 36015885 PMC9413329

[B8] Baret F. Guyot G. (1991). Potentials and limits of vegetation indices for lai and apar assessment. Remote Sens. Environ. 35, 161–173. doi: 10.1016/0034-4257(91)90009-U

[B9] Barreto A. Yamati F. R. I. Varrelmann M. Paulus S. Mahlein A. (2023). Disease incidence and severity of cercospora leaf spot in sugar beet assessed by multispectral unmanned aerial images and machine learning. Plant Dis. 107, 188–200. doi: 10.1094/PDIS-12-21-2734-RE 35581914

[B10] Cabezas J. Galleguillos M. Perez-Quezada J. F. (2016). Predicting vascular plant richness in a heterogeneous wetland using spectral and textural features and a random forest algorithm. IEEE Geosci. Remote Sens. Lett. 13, 646–650. doi: 10.1109/LGRS.2016.2532743

[B11] Chen D. Zhang F. Tan M. L. Chan N. W. Shi J. Liu C.. (2022). Improved na+ estimation from hyperspectral data of saline vegetation by machine learning. Comput. Electron. Agric. 196, 106862. doi: 10.1016/j.compag.2022.106862

[B12] Da Luz A. G. Bleninger T. B. Polli B. A. Lipski B. (2022). Spatio-temporal variation of aquatic macrophyte cover in a reservoir using landsat images and google earth engine. Rbrh-Revista Bras. Recursos Hidricos 27, 1–17. doi: 10.1590/2318-0331.272220220074

[B13] Datt B. McVicar T. R. Van Niel T. G. Jupp D. Pearlman J. S. (2003). Preprocessing eo-1 hyperion hyperspectral data to support the application of agricultural indexes. IEEE Trans. On Geosci. Remote Sens. 41, 1246–1259. doi: 10.1109/TGRS.2003.813206

[B14] Ehammer A. Fritsch S. Conrad C. Lamers J. Dech S. (2010). “Statistical derivation of fpar and lai for irrigated cotton and rice in arid Uzbekistan by combining multi-temporal rapideye data and ground measurements,” in REMOTE SENSING FOR AGRICULTURE, ECOSYSTEMS, AND HYDROLOGY XII (SPIE), vol. 9. (Toulouse, FRANCE), 7824. doi: 10.1117/12.864796

[B15] Gamon J. A. Surfus J. S. (1999). Assessing leaf pigment content and activity with a reflectometer. New Phytol. 143, 105–117. doi: 10.1046/j.1469-8137.1999.00424.x

[B16] Haboudane D. Miller J. R. Pattey E. Zarco-Tejada P. J. Strachan I. B. (2004). Hyperspectral vegetation indices and novel algorithms for predicting green lai of crop canopies: modeling and validation in the context of precision agriculture. Remote Sens. Environ. 90, 337–352. doi: 10.1016/j.rse.2003.12.013

[B17] Han P. Zhai Y. Liu W. Lin H. An Q. Zhang Q.. (2023). Estimating soil salinity using multiple spectral indexes and machine learning algorithm in songnen plain, China. IEEE J. Selected Topics Appl. Earth Observations Remote Sens. 16, 7041–7050. doi: 10.1109/JSTARS.2023.3274579

[B18] Hancock D. W. Dougherty C. T. (2007). Relationships between blue- and red-based vegetation indices and leaf area and yield of alfalfa. Crop Sci. 47, 2547–2556. doi: 10.2135/cropsci2007.01.0031

[B19] He Y. Guo X. Wilmshurst J. F. (2007). Comparison of different methods for measuring leaf area index in a mixed grassland. Can. J. Plant Sci. 87, 803–813. doi: 10.4141/CJPS07024

[B20] Ihuoma S. O. Madramootoo C. A. (2019). Sensitivity of spectral vegetation indices for monitoring water stress in tomato plants. Comput. Electron. Agric. 163, 104860. doi: 10.1016/j.compag.2019.104860

[B21] Jeon H. Oh S. (2020). Hybrid-recursive feature elimination for efficient feature selection. Appl. Sciences-Basel 10, 3211. doi: 10.3390/app10093211

[B22] Jiang Z. Huete A. R. Didan K. Miura T. (2008). Development of a two-band enhanced vegetation index without a blue band. Remote Sens. Environ. 112, 3833–3845. doi: 10.1016/j.rse.2008.06.006

[B23] Jordan C. F. (1969). Derivation of leaf-area index from quality of light on the forest floor. Ecology 50, 663–666. doi: 10.2307/1936256

[B24] Jr. Hunt E. R. Daughtry C. S. T. Eitel J. U. H. Long D. S. (2011). Remote sensing leaf chlorophyll content using a visible band index. Agron. J. 103, 1090–1099. doi: 10.2134/agronj2010.0395

[B25] Khaki S. Wang L. Archontoulis S. V. (2020). A cnn-rnn framework for crop yield prediction. Front. Plant Sci. 10. doi: 10.3389/fpls.2019.01750 PMC6993602 32038699

[B26] King A. D. Donat M. G. Fischer E. M. Hawkins E. Alexander L. V. Karoly D. J.. (2015). The timing of anthropogenic emergence in simulated climate extremes. Environ. Res. Lett. 10, 94015. doi: 10.1088/1748-9326/10/9/094015

[B27] Koc A. Odilbekov F. Alamrani M. Henriksson T. Chawade A. (2022). Predicting yellow rust in wheat breeding trials by proximal phenotyping and machine learning. Plant Methods 18, 30. doi: 10.1186/s13007-022-00868-0 35292072 PMC8922805

[B28] Kohavi R. John G. H. (1997). Wrappers for feature subset selection. Artif. Intell. 97, 273–324. doi: 10.1016/S0004-3702(97)00043-X

[B29] Leo S. De Antoni Migliorati M. Grace P. R. (2021). Predicting within-field cotton yields using publicly available datasets and machine learning. Agron. J. 113, 1150–1163. doi: 10.1002/agj2.20543

[B30] Lesk C. Anderson W. Rigden A. Coast O. Jaegermeyr J. McDermid S.. (2022). Compound heat and moisture extreme impacts on global crop yields under climate change. Nat. Rev. Earth Environ. 3, 872–889. doi: 10.1038/s43017-022-00368-8

[B31] Li Z. Chen Z. Cheng Q. Duan F. Sui R. Huang X.. (2022). Uav-based hyperspectral and ensemble machine learning for predicting yield in winter wheat. Agronomy-Basel 12, 10202. doi: 10.3390/agronomy12010202

[B32] Li Z. Li Z. Fairbairn D. Li N. Xu B. Feng H.. (2019). Multi-luts method for canopy nitrogen density estimation in winter wheat by field and uav hyperspectral. Comput. Electron. Agric. 162, 174–182. doi: 10.1016/j.compag.2019.04.005

[B33] Li Y. Xu Z. Hao Z. Yao X. Zhang Q. Huang X.. (2023). A comparative study of the performances of joint rfe with machine learning algorithms for extracting moso bamboo (phyllostachys pubescens) forest based on uav hyperspectral images. Geocarto Int. 38, 2207550. doi: 10.1080/10106049.2023.2207550

[B34] Li Z. Zhou X. Cheng Q. Fei S. Chen Z. (2023). A machine-learning model based on the fusion of spectral and textural features from uav multi-sensors to analyse the total nitrogen content in winter wheat. Remote Sens. 15, 2152. doi: 10.3390/rs15082152

[B35] Liu J. Si Z. Wu L. Shen X. Gao Y. Duan A. (2023). High-low seedbed cultivation drives the efficient utilization of key production resources and the improvement of wheat productivity in the north China plain. Agric. Water Manage. 285, 108357. doi: 10.1016/j.agwat.2023.108357

[B36] Lo Y. Fu L. Lu T. Huang H. Kong L. Xu Y.. (2023). Medium-sized lake water quality parameters retrieval using multispectral uav image and machine learning algorithms: a case study of the yuandang lake, China. Drones 7, 244. doi: 10.3390/drones7040244

[B37] LUO Y. XU J. YUE W. CHEN W. (2006). A comparative study of extracting urban vegetation information by vegetation indices from thematic mapper images. Remote Sens. Technol. Appl. 21, 212–219. doi: 10.1007/s11769-006-0026-1

[B38] Ma Y. Jiang Q. Meng Z. Liu H. Jiao W. (2018). Black soil organic matter content estimation using hybrid selection method based on rf and gabpso. Spectrosc. Spectral Anal. 38, 181–187. doi: 10.3964/j.issn.1000-0593(2018)01-0181-07

[B39] Ma J. Liu B. Ji L. Zhu Z. Wu Y.. (2023). Field-scale yield prediction of winter wheat under different irrigation regimes based on dynamic fusion of multimodal uav imagery. Int. J. Appl. Earth Observation Geoinformation 118, 103297. doi: 10.1016/j.jag.2023.103292

[B40] Main R. Cho M. A. Mathieu R. O’Kennedy M. M. Ramoelo A. Koch S. (2011). An investigation into robust spectral indices for leaf chlorophyll estimation. Isprs J. Photogrammetry Remote Sens. 66, 751–761. doi: 10.1016/j.isprsjprs.2011.08.001

[B41] Mao J. Xu W. Yang Y. Wang J. Yuille A. L. (2014). Explain images with multimodal recurrent neural networks. Arxiv 10, 48550. doi: 10.48550/arXiv.1410.1090

[B42] Marques Ramos A. P. Osco L. P. Garcia Furuya D. E. Goncalves W. N. Santana D. C. Ribeiro Teodoro L.P.. (2020). A random forest ranking approach to predict yield in maize with uav-based vegetation spectral indices. Comput. Electron. Agric. 178, 105791. doi: 10.1016/j.compag.2020.105791

[B43] Mihalache S. Burileanu D. (2022). Using voice activity detection and deep neural networks with hybrid speech feature extraction for deceptive speech detection. Sensors 22, 1228. doi: 10.3390/s22031228 35161973 PMC8839638

[B44] Murata R. Okubo F. Minematsu T. Taniguchi Y. Shimada A. (2023). Recurrent neural network-fitnets: improving early prediction of student performanceby time-series knowledge distillation. J. Educ. Computing Res. 61, 639–670. doi: 10.1177/07356331221129765

[B45] Nguyen C. Sagan V. Skobalski J. Severo J. I. (2023). Early detection of wheat yellow rust disease and its impact on terminal yield with multi-spectral uav-imagery. Remote Sens. 15, 133301. doi: 10.3390/rs15133301

[B46] Ou Q. Lei X. Shen C. (2019). Individual tree diameter growth models of larch-spruce-fir mixed forests based on machine learning algorithms. Forests 10, 20187. doi: 10.3390/f10020187

[B47] Pocas I. Rodrigues A. Goncalves S. Costa P. M. Goncalves I. Pereira L.S.. (2015). Predicting grapevine water status based on hyperspectral reflectance vegetation indices. Remote Sens. 7, 16460–16479. doi: 10.3390/rs71215835

[B48] Raper T. B. Varco J. J. (2015). Canopy-scale wavelength and vegetative index sensitivities to cotton growth parameters and nitrogen status. Precis. Agric. 16, 62–76. doi: 10.1007/s11119-014-9383-4

[B49] Roujean J. L. Breon F. M. (1995). Estimating par absorbed by vegetation from bidirectional reflectance measurements. Remote Sens. Environ. 51, 375–384. doi: 10.1016/0034-4257(94)00114-3

[B50] Sarkodie V. Y. O. Vasat R. Pouladi N. Sramek V. Sanka M. Fadrhonsova V.. (2023). Predicting soil organic carbon stocks in different layers of forest soils in the Czech Republic. Geoderma Regional 34, e00658. doi: 10.1016/j.geodrs.2023.e00658

[B51] Shafiee S. Mroz T. Burud I. Lillemo M. (2023). Evaluation of uav multispectral cameras for yield and biomass prediction in wheat under different sun elevation angles and phenological stages. Comput. Electron. Agric. 210, 107874. doi: 10.1016/j.compag.2023.107874

[B52] Su X. Wang J. Ding L. Lu J. Zhang J. Yao X.. (2023). Grain yield prediction using multi-temporal uav-based multispectral vegetation indices and endmember abundance in rice. Field Crops Res. 299, 108992. doi: 10.1016/j.fcr.2023.108992

[B53] Tran T. V. Reef R. Zhu X. (2022). A review of spectral indices for mangrove remote sensing. Remote Sens. 14, 194868. doi: 10.3390/rs14194868

[B54] Tucker C. J. (1979). Red and photographic infrared linear combinations for monitoring vegetation. Remote Sens. Environ. 8, 127–150. doi: 10.1016/0034-4257(79)90013-0

[B55] Tucker C. J. Jr. McMurtrey J. E. I. Fan C. J. (1979). Monitoring corn and soybean crop development with hand-held radiometer spectral data. Remote Sens. Environ. 8, 237–248. doi: 10.1016/0034-4257(79)90004-X

[B56] Wang X. Miao Y. Guan Y. Xia T. Lu J. Mulla D.J. (2016). “An evaluation of two active canopy sensor systems for non-destructive estimation of spring maize biomass,” in 2016 Fifth International Conference on Agro-Geoinformatics (Agro-Geoinformatics) (Tianjin, China: IEEE). 340–345. doi: 10.1109/Agro-Geoinformatics.2016.7577610

[B57] Wu H. Su X. Zhang G. Feng K. Liang Z. (2022). Statistical prediction of agricultural drought severity in China based on dry or hot events. Theor. Appl. Climatology 147, 159–171. doi: 10.1007/s00704-021-03797-5

[B58] Xiao W. Ye X. Zhu Z. Zhang Q. Zhao S. Chen D.. (2022). Evaluation of cadmium (cd) transfer from paddy soil to rice (oryza sativa l.) Using dgt in comparison with conventional chemical methods: derivation of models to predict cd accumulation in rice grains. Environ. Sci. and Pollu. Res. 27, 14953–14962. doi: 10.1007/s11356-020-07976-1 32062776

[B59] Xiao Y. Xue J. Zhang X. Wang N. Hong Y. Jiang Y.. (2022). Improving pedotransfer functions for predicting soil mineral associated organic carbon by ensemble machine learning. Geoderma 428, 116208. doi: 10.1016/j.geoderma.2022.116208

[B60] Yin Y. Jang-Jaccard J. Xu W. Singh A. Zhu J. Sabrina F.. (2023). Igrf-rfe: a hybrid feature selection method for mlp-based network intrusion detection on unsw-nb15 dataset. J. Big Data 10, 15. doi: 10.1186/s40537-023-00694-8

[B61] Zhang X. Xue J. Xiao Y. Shi Z. Chen S. (2023). Towards optimal variable selection methods for soil property prediction using a regional soil vis-nir spectral library. Remote Sens. 15, 2. doi: 10.3390/rs15020465

[B62] Zhang C. Yang K. Li Y. Cheng F. Rong K. (2020). Spectral characteristics and the study of pollution degree of maize leaves under copper and lead stress. J. Indian Soc. Remote Sens. 48, 21–33. doi: 10.1007/s12524-019-01055-w

[B63] Zheng H. Cheng T. Zhou M. Li D. Yao X. Tian Y.. (2019). Improved estimation of rice aboveground biomass combining textural and spectral analysis of uav imagery. Precis. Agric. 20, 611–629. doi: 10.1007/s11119-018-9600-7

[B64] Zhou Y. Zhao X. Guo X. Li Y. (2022). Mapping of soil organic carbon using machine learning models: combination of optical and radar remote sensing data. Soil Sci. Soc. America J. 86, 293–310. doi: 10.1002/saj2.20371

